# Supplementary material for: The interactome of intact mitochondria by cross-linking mass spectrometry provides evidence for coexisting respiratory supercomplexes
Source: Mol Cell Proteomics. 2017 Dec 8;17(2):216–32. doi: 10.1074/mcp.RA117.000470 (PMC5795388; doi:10.1074/mcp.RA117.000470)
Supplement: Supplemental Data [file supp_RA117.000470_133922_0_supp_23978_fzffwf.zip › spectra_annotation/mito_DR_spectra_annotation/171-1-16-1-7-1.pdf]

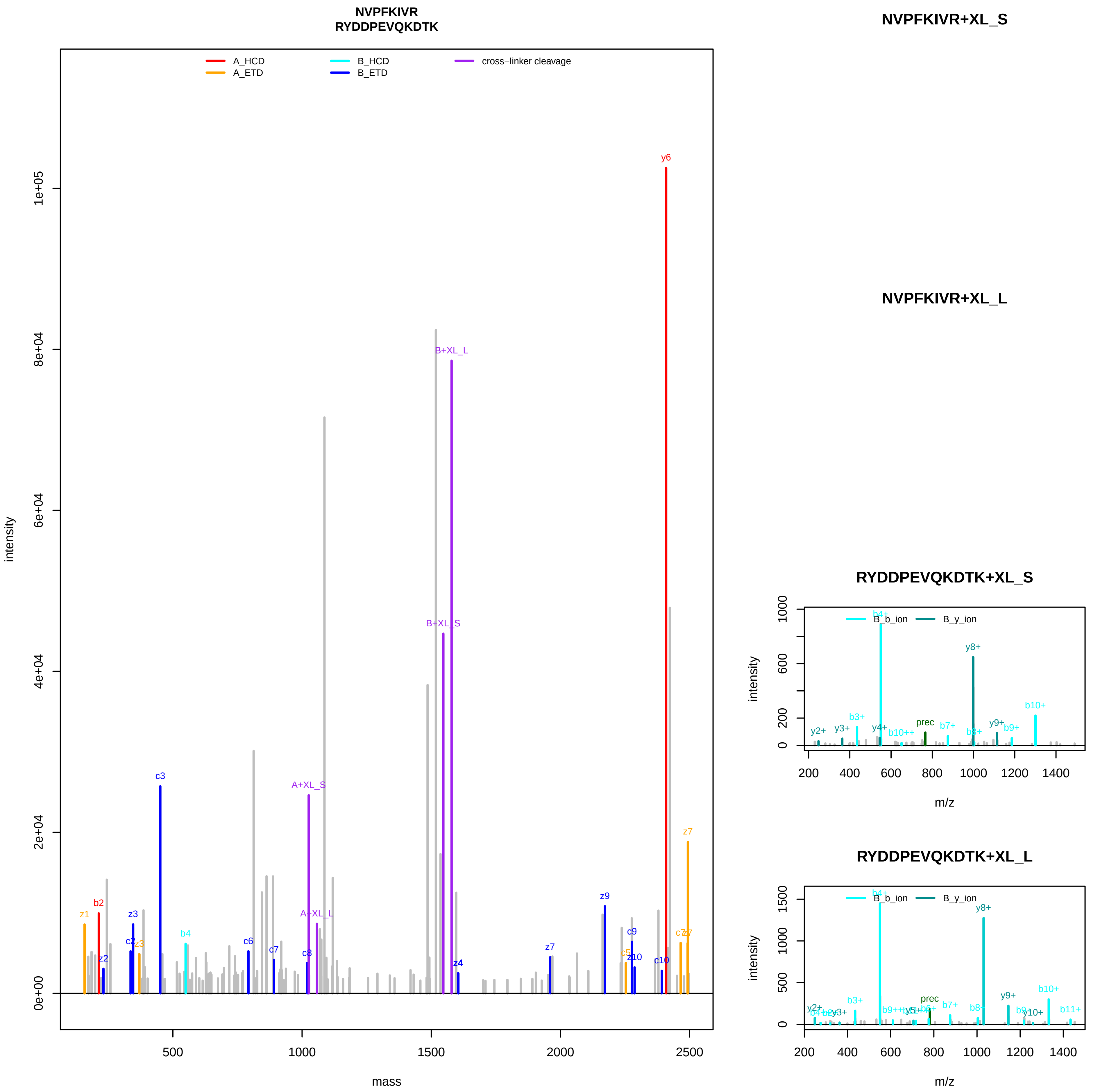
NVPFKIVR
RYDDPEVQKDTK
A_HCD
A_ETD
B_HCD
B_ETD
cross−linker cleavage
intensity
mass
y6
B+XL_L
B+XL_S
A+XL_S
A+XL_L
c3
z1
b2
z2
c2
z3
b4
c6
c7
c8
z4
z7
z9
c9
z10
c5
c10
c7
z7
NVPFKIVR+XL_S
NVPFKIVR+XL_L
RYDDPEVQKDTK+XL_S
RYDDPEVQKDTK+XL_L
B_b_ion
B_y_ion
m/z
prec
y8+
b10+
b3+
b4+
y9+
b9+
b7+
b8+
y2+
y3+
y4+
b10++
y10+
b11+
b9++
y5+
b6+
